# Quality of life and physical activity among older adults living in institutions compared to the community

**DOI:** 10.4102/sajp.v73i1.342

**Published:** 2017-07-28

**Authors:** Lesego M. Ramocha, Quinette A. Louw, Muziwakhe D. Tshabalala

**Affiliations:** 1 Division of Clinical Epidemiology and Biostatistics, Department of Global Health, Stellenbosch University, South Africa; 2 Division of Physiotherapy, Department of Interdisciplinary Health Sciences, Stellenbosch University, South Africa; 3 Department of Physiotherapy, School of Health Care Sciences, Sefako Makgatho Health Sciences University, South Africa

## Abstract

**Background:**

The environment of older adults plays an important role in their well-being. It influences their quality of life and physical activity level. In South Africa, there is a dearth of literature concerning this issue.

**Methods:**

An analytic cross-sectional sample of 80 older adults living in old age homes and the community was compared in terms of level of physical activity and quality of life. The study was conducted in Soweto, Johannesburg. A computer-generated random sample of older adults aged 60 years and above participated. The Physical Activity Scale for the Elderly (PASE) and RAND 36 questionnaires were used for data collection. Descriptive statistics were used to describe the sample. Unpaired *t*-tests, Pearson’s correlation coefficient and chi-squared test explored the differences and associations between institutionalised and community living older adults.

**Results:**

Quality of life in old age home dwellers (*M* = 68.53 ± 19.55) was significantly lower (*p* = 0.025) than in community dwellers (*M* = 77.74 ± 16.25). The mean physical activity score was also significantly (*p* = 0.000) lower in old age home dwellers (*M* = 20.18 ± 24.52) compared with community dwellers (*M* = 190.31 ± 82.81).

**Conclusion:**

Older adults who live in the community have a higher quality of life and physical activity levels compared with those who live in institutions (old age homes).

## Introduction

*Geriatric* is a medical term that encompasses the health of elderly patients. While no specific age has been set for defining older adults, the general consensus considers the age of 60 years as a cut off (WHO 2015). Worldwide, the number of older adults aged 60 years and above is projected to increase from 841 million people in 2013 to more than 2 billion in 2050 (UN 2013). Older adults are projected to surpass the number of children for the first time in 2047. Similar trends have been projected for Africa. The World Health Organization (WHO) reports that sub-Saharan Africa’s older adult population will increase from 43 million in 2010 to 67 million in 2025, peaking at 163 million by 2050 (WHO 2013). South Africa is among the countries in Africa that has an increasingly ageing population. Joubert and Bradshaw (2005) estimate that the population will continue ageing over the next two decades such that older people will account for 10.5% of the total South African population and will number 5 million by 2025.

As a person grows old, there is a level of vulnerability that develops because of physiological changes, unemployment, poor health, financial challenges and neglect by society (Lakshmi & Roopa 2013). An Asian study reported that older adults who live alone are more prone to depression and social isolation (Lim & Kua 2011). In contrast, community living older adults have been shown to have better outcomes. A review based on three small European trials has shown that living in the community may reduce the chance of premature death (Shepperd, Wee & Straus 2011). The same study also showed that living in the community may be related to improved quality of life, function or cognitive abilities (such as mental alertness and thinking) compared with living in an institution like an old age home.

Walking is the most common physical activity performed by older adults (Australian Bureau of Statistics 2011). In the United States, older adults commonly engage in low-intensity activities such as walking, gardening or golf, rather than running, aerobics or team sports (DiPietro 2001). Increased physical activity in older adults has been demonstrated to be predictive of better future physical function and self-efficacy (Jack et al. 2008). The same study further highlighted the beneficial impact of physical activity on the mental health status of older adults. In addition, Rejeski, Brawley and Shumaker (1996) have demonstrated that physical activity improves the overall quality of life despite age, activity status or the health of an old person.

There is limited research in Africa concerning older adults in general, and particularly on the effect of institutionalised care. In a report on the quality of life of older adults living in institutions and homes in Zimbabwe, Nyanguru (1990) observed that institutions and homes caring for older adults are predominantly located in urban areas, and that there were more homes for white than black Africans. Citing the general lack of pensions and retirement plans, Nyanguru proposed that black Africans enter old age homes largely because of being destitute in contrast to their white peers for whom institutional care was planned and better financed.

Old age homes in South Africa arose as a response to several social problems including loneliness, economic and housing problems, and lack of family and other support systems for older adults (Perold & Muller 2011). Care of older adults will assume increasing public health and policy importance as the number of old people continues to grow, both globally and locally (UN 2013). A prominent question centres on different modes of care: community living versus institutional care. In this context, we set out to investigate the differences between these modes of care by comparing the quality of life and physical activity of older adults who are living in institutions and those living in the community (community dwellers).

## Methodology

The study was conducted in Soweto, Johannesburg. An analytic cross-sectional study was conducted to measure the level of quality of life and physical activity among older adults living in the community compared with those living in old age homes. Validated questionnaires RAND 36 and the Physical Activity Scale for the Elderly (PASE) were used as outcome measures. The Mini Mental State Examination (MMSE) was used to screen participants for inclusion in the study.

### Sample

The inclusion criteria for the study were men and women at least 60 years old, of all races and living in either old age homes or the community. Participants with mental disability were excluded from the study. A total of 80 participants (40 in each group) was selected. Two stage sampling was done: convenient sampling of the sites and random sampling of the participants from the study site. A sampling frame was constructed with all the eligible participants from all the study sites.

We conducted a post hoc sample size calculation using our study’s data (because of a lack of published African data) to determine the statistical power of the study. Using G-power version 3.1.7 and our main finding related to quality of life of home dwellers (*M* = 68.5 ± 19.5) and for community dwellers (*M* = 77.7 ± 16.2), a medium effect size (*d* = 0.5) and a probability of 0.05, the post hoc calculation showed that the power of our study was 60%. This implies a 60% likelihood to detect a difference between groups and if the study is to be repeated, 6 out of 10 studies will yield a similar difference. As we have detected significant differences of the main outcomes (because of large group differences), we are not concerned about type II errors (differences undetected because of sample size).

The Department of Social Development gave us a list of all the Soweto old age homes and the luncheon gatherings for the elderly who live in the community. The luncheon gatherings and old age homes are projects that are funded by the Department of Social Development. Of the 10 luncheon gatherings in different communities, only four were included for recruitment of the community dwelling older adults. Some luncheon gatherings no longer existed while others had changed their contact numbers. The Soweto communities included and their respective numbers of older adults were: Kgotso Uxolo (35), Bambanani (170), Naledi ya Meso (50) and Noordecesig (21). Three old age homes were identified from which institutional older adults were recruited. All three old age homes in Soweto were included. These old age homes and their respective numbers of older adults were: Ephraim Zulu (86), Soweto Home of The Aged (112) and Meadowlands Care for the Aged (65).

### Instruments

The RAND 36 (Ware & Sherbourne 1992) and the PASE questionnaires were used to collect data. The RAND 36 tool measures self-perceived quality of life. It uses eight domains: physical functioning, bodily pain, limitation because of physical health problems, role limitation because of personal or emotional problems, emotional well-being, social functioning, energy or fatigue and general health perception. Each domain is scaled from 0 to 100. Domain scores are totalled and averaged to give the overall RAND 36 total score, also scaled from 0 to 100. Higher scores indicate good quality of life. This reliable and validated tool has been used in South African studies (Moller & Smit 2004; Van Rensburg et al. 2010). Although the RAND 36 tool has been widely used in South Africa, the values of its validity and reliability measurement in a South African context have not yet been explored. The RAND 36 and the SF-36 have the same set of items but have differences in the scoring. An evaluation of the SF-36 conducted in Ghana showed that the reliability for the eight scales of the SF-36 was 0.82 (Cronbach’s alpha > 0.70), and the standardised alpha coefficient was 0.86 (Faustina & Allan 2014).

The PASE (New England Research Institute 1991) is an interviewer or self-administered recall questionnaire designed to assess physical activity among older adults. The PASE score is derived from predetermined weights and frequency values for each of the 12 activity items. Scores range from 0 to 400. The tool is designed to assess household, occupational and leisure activity items. Sociodemographic characteristics which include gender, age, race, marital status, source of income and type of home were added to the original questionnaire. A test-retest reliability was assessed in a South African population in a period of 3–7 weeks and the value was 0.75 (95% CI = 0.69–0.80). The reliability value for a questionnaire that was answered through a mail administration was (*r* = 0.84) higher compared with telephonic administration (*r* = 0.68). Construct validity was shown by correlating PASE scores with health status and physiologic measures and the results were as follows: grip strength (*r* = 0.37), static balance (*r* = +0.33), leg strength (*r* = 0.25) and negatively correlated with resting heart rate (*r* = -0.13), age (*r* = -0.34) and perceived health status (*r* = -0.34), and overall Sickness Impact Profile score (*r* = -0.42). The study showed that PASE is a trustworthy tool for the evaluation of physical activity in older adults (Washburn et al. 1993).

The questionnaires were translated to local languages (Setswana and isiZulu) and pretested for face validity and content validity. The translation and back-translation were done by native Setswana and isiZulu speakers who are proficient and fluent in writing and speaking the languages. Test-retest reliability of the translated questionnaires was done by professional translators 7 days before the first administration of the tools to ensure fidelity of translation. The pilot testing revealed adequate translation with no further corrections made on the tool. The questionnaire was interviewer-administered.

The MMSE is a validated reliable tool for assessing cognitive function of the community dwelling, hospitalised and institutionalised elderly. There are 11 questions with a maximum score of 30. A score of 23 or lower indicates cognitive impairment. The MMSE takes about 5–10 min to administer. This tool has been widely used in a South African population (Ganasen et al. 2008; Van Scalkwyk, Botha & Seedat 2012). This tool has been used in older people and shows high reliability and validity (Keskinoglu et al. 2009). The tool has a cut off of 22/23 with a good sensitivity, specificity and positive likelihood ratio of 90.91, 97.01 and 30.3, respectively. The uneducated elderly cut off point is 18/19 with a sensitivity score of 82.71, specificity 92.32 and positive likelihood ratio at 10.7. Kappa value of the test is in perfect agreement, an interrater of 1.00. The correlation results between intrarater and interrater test-retest in educated elderly subjects are 0.966 (*p* = 0.000); 0.85 (*p* = 0.000), respectively, while in uneducated elderly the result is 0.98 (*p* = 0.000); 0.93 (*p* = 0.000), respectively (Keskinoglu et al. 2009). This result shows that the MMSE is a highly reliable tool that can be used in older people.

### Statistical analysis

Questionnaire gathered data were transcribed onto a Microsoft Excel spreadsheet, double checked for errors and then imported into the Statistical Package for Social Sciences (SPSS, version 23) for analysis. Means, standard deviations and frequencies were described. The chi-square test was also used to determine associations between the study groups. Data were tested for normality of distribution using the Shapiro-Wilks test. Differences in the quality of life and physical activity between the two study groups were explored using unpaired *t*-tests, while potential associations between quality of life, physical activity and type of dwelling were explored using Pearson’s correlation test. All tests were considered statistically significant at a *p*-level of 0.05.

### Ethical consideration

Ethical clearance was obtained from the University of Stellenbosch Ethics Committee (reference no. S15/05/106). Additional permission was sought and obtained from the Department of Social Development. Participation in the study was voluntary and participants were able to withdraw at any point in the study without giving a reason. Anonymity was maintained throughout and the questionnaires did not collect identifying information from participants. All participants signed informed consent prior to participation.

## Results

### Demographic profile

Baseline demographic characteristics of the study participants are displayed in Table 1. The chi-square test was used for analysis. However, of note was the lack of male community living older adults, in contrast to the near equal gender distribution among the home-dwelling older adults (female 42.5% vs male 57.5%). There were no statistically significant differences in educational attainment or marital status.

**TABLE 1:** Distribution of sociodemographics of old age home and community dwellers.

Sociodemographics	Old-aged home		Community dwelling		*Χ*^2^ value	*p*
	
	*n*	%	*n*	%		
**Age**						
60–65	9	22.5	7	17.5	33.4	0.22
66–70	11	27.5	6	15.0		
71–75	12	30.0	10	25.0		
76–80	5	12.5	6	15.0		
81–85	2	5.0	8	20.0		
86–90	1	2.5	3	7.5		
**Gender**						
Female	17	42.5	40	100	21.58	0.001
Male	23	57.5	0	0		
**Marital status**						
Single	9	22.5	5	12.5	5.30	0.15
Married	8	20.0	5	12.5		
Divorced	5	12.5	2	5.0		
Widowed	18	45.0	28	70.0		
**Educational status**						
Never	7.5	7.5	2	5.3	4.30	0.23
Primary	17.5	17.5	5	12.5		
Secondary	70	70.0	25	62.5		
Tertiary	5	5.0	8	20.0		

*n*, number of participants; %, percentage; *Χ*^2^, chi-square test at *p* < 0.05.

### Quality of life and physical activity profiles

The eight domains of the RAND 36 quality of life questionnaires were explored (Table 2). Overall, community dwelling older adults study participants reported higher levels of quality of life compared with their old-age-home-dwelling counterparts: *M* = 77.74, (SD = ±16.25) versus *M* = 68.53 (SD = ±19.55), respectively, with a *p*-value of 0.025. Within-domain comparisons demonstrated significant differences between the two study groups (*p*-values of 0.004 and 0.001, respectively). Quality of life and levels of physical activity were higher in the community dwellers compared with older people living in old age homes.

**TABLE 2:** RAND 36 domains and PASE scores (*n* = 80).

RAND 36 domain	Old age home		Community		*t*	df	*p*
	
	*M*	± SD	*M*	± SD			
Physical functioning	74.7	29.6	81.1	22.9	1.1	78	0.28
Physical health	61.2	47.3	68.1	44.2	0.6	78	0.50
Emotional problems	59.1	46.2	74.1	42.3	1.1	78	0.13
Energy or fatigue	66.3	20.5	79.5	19.1	2.9	78	0.001*
Emotional well-being	73.9	19.0	86.8	13.1	3.53	78	0.001*
Social functioning	68.9	21.4	77.1	20.5	1.7	78	0.08
Bodily pain	66.7	28.9	73.8	26.4	1.1	78	0.25
General health	66.1	20.0	73.0	18.9	1.6	78	0.09
Total RAND 36	68.5	19.5	77.7	16.2	2.2	78	0.02*
PASE score	20.18	24.52	190.31	82.81	12.46	78	0.00

*P*-value is the statistical difference between old age home and community living at *p* < 0.05.

*M*, represents means; ± SD, represents standard deviations; *t* means unpaired *t*-tests; df is the degree of freedom.

* , *p* < 0.05

Similarly, there were statistically significant differences between the PASE scores. The mean score for the community dwellers was significantly better (*p* < 0.000) for community dwellers compared with old age home dwellers *M* = 20.18 (± 24.52) *p* < 0.00.

Table 3 below indicates the association of quality of life and physical activity for the old age home and community dwellers. The social function domain demonstrated a weak, positive correlation of *r* = 0.318 that is statistically significant at *p* = 0.046. The remaining domains were found to have weak correlations and did not show a statistically significant difference.

**TABLE 3:** Pearson’s correlation of RAND 36 domains and PASE score of old age home and community dwellers (*n* = 80).

RAND 36 domains and PASE score	Correlation	*p*
Physical function	−0.12	0.42
Physical health	−0.05	0.74
Emotional problems	0.14	0.36
Energy or fatigue	0.01	0.92
Emotional well-being	0.00	0.95
Social functioning	0.31	0.04*
Bodily pain	0.02	0.86
General health	0.20	0.20

**Total RAND 36**	0.09	0.55
**PASE score**	−0.20	0.17

*n*, number of participants and *p*-value is the statistical difference at *p* (2-tailed) < 0.05.

PASE, Physical Activity Scale for the Elderly.

* , *p* < 0.05

Table 4 below depicted the contrast in the level of physical activity among the old age home dwellers when compared with community dwellers. The chi-square test showed that physical activity level and quality of life were significantly better in community dwellers (*Χ*^2^ = 65.33; *p* < 0.05) compared with older people living in old age homes.

**TABLE 4:** The comparison of activity level of old age home vs. community dwellers.

Activity level	Old age home		Community dwellers		*Χ*^²^ value	*p*
	
	*n*	%	*n*	%		
Vigorous PA	0	0.0	32	80.0	65.33	0.00
Moderate PA	5	12.5	7	17.5		
Minimal PA	3	7.5	1	2.5		
No PA	32	80.0	0	0.0		

*n*, number of participants; %, percentage; PA, physical activity.

The number of community dwellers who participate in vigorous activities was very high with an 80% score. Old age home dwellers reported very high percentages of inactivity. The old age home dwellers participated in minimal or no physical activity. It is important to note that all of the community dwellers in the study participated in some physical activity.

## Discussion

The community dwellers had a significantly higher quality of life in comparison to the old age home dwellers. Out of the eight domains of the RAND 36, two domains, energy or fatigue and emotional well-being showed a statistically significant difference.

These findings suggest that the quality of life in older adults may be improved by programmes that improve emotional well-being and energy levels. A similar strategy was applied in an 18-month follow-up of community dwelling older adults (Phillips, Wójcicki & McCauley 2013). Reports from India by Mathew, George and Paniyad (2009) also corroborate our findings of lower quality of life among institutionalised older adults. Contrary findings have been reported by Lakshmi and Roopa (2013) who reported higher quality of life in institutionalised older adults compared with non-institutionalised older adults. The perception of quality of life is derived from a range of constructs which often differ between cultures. In the study by Lakshmi and Roopa, it is unclear which factors played a role in the improved quality of life among older people living in India. Differences in the constructs that inform quality of life measured between our study and the study by Lakshmi and Roopa may explain the differences in study outcomes.

Most old age home dwellers were less active physically in comparison with community dwellers. Community living is associated with higher PASE score in Canada (Chad et al. 2005). The positive relationship between physical activity and quality of life is noted in published reports (Acree et al. 2006; Drewnowski & Evans 2001; Strawbridge et al. 2002) A systematic review (Vagetti et al. 2014) which aimed at determining associations between physical activity and domains of quality of life showed a positive association between physical activity and quality of life in the elderly.

The physical and physiological benefits of physical activity of engaging in regular exercise assist the elderly with functions such as household activities. Consequently, independence and mental health are enhanced and these lead to improved quality of life (Phillips et al. 2013; Vagetti et al. 2014). Of note, participant characteristics associated with high PASE scores in our study were being married, community living and higher levels of education. However, we noted that more old age home dwellers were married but not physically active. The majority of participants were female older than 65 years and were without partners. Most adults in our study attended school up to secondary level, in keeping with nationally reported low educational attainments of adults in South Africa (South Africa Statistics 2011).

The WHO (2016) recommends that older adults should incorporate at least 150 min of moderate to intense activities throughout the week. The community dwellers in our study surpassed this guideline for healthy living. Although this is very reassuring for the health of older people living in our communities, further studies are required to determine the predictors of physical activity in older adults living in the community in South Africa.

Physiotherapists play an integral role in promoting physical activity. Physical activities promote independence, participation in activities of daily living and result in good mental and physical health of the elderly (Gislaine et al. 2014). Factors such as family members and spousal support also play an important role to ensure that older people remain physically active for as long as possible (Lim & Kua 2011). Intrinsic factors such as self-will drive to stay active are also crucial (Gislaine et al. 2014). Educating the elderly on the importance of physical activity and its impact on quality of life is strongly recommended to physiotherapists and caregivers. The findings of this study further suggest that physiotherapists should introduce interventions to enable institutionalised elderly persons to be more physically active.

### Limitations

The study data were collected through questionnaires with self-reporting, rendering data liable to recall bias. Under-representation of male participants in the community dwelling group severely limits the generalisability of our data. Inferences to the wider general older adults are further restricted by our exclusion of those with mental disorders or illness. Mental illness, notably dementia, is increasingly prevalent among older adults who in turn may represent a sub-population with unique care needs. Given the post hoc power of the study (60%), further studies are needed to refute or corroborate our findings.

## Conclusion

The older adults who live in the community had a higher level of quality of life and physical activity compared with those living in old age homes. Notwithstanding the study’s limitations, our results suggest that older adults are better motivated to be physically active when they live in the community than in old age homes. These findings also imply that there should be greater emphasis on physical activity in old age homes. Future studies should include more men to facilitate generalisability of findings.
